# Moral values of Dutch physicians in relation to requests for euthanasia: a qualitative study

**DOI:** 10.1186/s12910-022-00834-4

**Published:** 2022-09-20

**Authors:** Marjanne van Zwol, Fijgje de Boer, Natalie Evans, Guy Widdershoven

**Affiliations:** Department of Ethics, Law and Medical Humanities, Amsterdam UMC, VU University, Verversstraat 153, 1011 HZ Amsterdam, The Netherlands

**Keywords:** Autonomy, Euthanasia, Medical ethics, Primary health care, Value of life

## Abstract

**Background:**

In the Netherlands, patients have the legal right to make a request for euthanasia to their physician. However, it is not clear what it means in a moral sense for a physician to receive a request for euthanasia. The aim of this study is to explore the moral values of physicians regarding requests for euthanasia.

**Methods:**

Semi-structured interviews were conducted with nine primary healthcare physicians involved in decision-making about euthanasia. The data were inductively analyzed which lead to the emergence of themes, one of which was about values regarding end-of-life decisions.

**Results:**

Four clusters of values related to euthanasia requests are described: values related to 1) the patient; 2) the family; 3) the physician; and 4) life and death. The data show that the participants value patient autonomy as a necessary but not sufficient condition for meeting a euthanasia request. A good relationship with the patient and the family are important. For the physician, the values physician autonomy, responsibility, understanding the patient and relief of suffering play a role. Life as an intrinsic good and a peaceful death are also important values.

**Conclusion:**

This study shows that next to patient autonomy and the relationship with the patient and the family, it is important for the participants to act in accordance with their professional values and to do justice to values related to life and death. The awareness of going against the intrinsic value of life is crucial, even if performing euthanasia may result in a feeling of relief or gratitude afterwards.

## Background

In the Netherlands, euthanasia has been the subject of debate for decades and is the subject of intense media focus. In the media, patient autonomy is a central issue [13]. Participants in the public debate argue that patients should be able to decide about their time of death, referring to the concept of autonomy in the sense of self-determination. This concept of autonomy entails that a person should be able to shape her life according to her own values and decisions, and not be dependent on others. In the words of Beauchamp and Childress [1]: “The principle of autonomy … asserts a right of non-interference and correlatively an obligation not to constrain autonomous actions.”.

Euthanasia is legally defined as "… the physician who, subject to the due care requirements of the law, terminates a life on request or provides assistance with suicide …" [14]. The due care requirements are: a) the request of the patient is voluntary and considered; b) the patient's suffering is hopeless and unbearable; c) the patient has been informed about his situation; d) there is no reasonable other solution; e) a second doctor has been consulted; and f) the termination of life has been carefully carried out (Regional Euthanasia Review [10].

Only physicians are allowed to legally perform euthanasia**.** The patient has the right to ask the physician for euthanasia and the physician has to take the request seriously. The physician, however, is not legally obliged to perform euthanasia. Conducting euthanasia or abstaining from it is a personal decision of the physician. The Royal Dutch Medical Association [14] states on their website: "Fundamental objections from physicians to euthanasia and suicide assistance must be respected".

Although approximately 4% of deaths in the Netherlands in 2019 are due to euthanasia [11] and most general practitioners (GPs) will receive a euthanasia request during their career, there is not much qualitative research concerning physicians’ personal views and values regarding euthanasia decision-making. Interview studies with Dutch GPs [6], [15] have found that physicians’ personal values do indeed influence decisions to grant euthanasia requests.

When a physician receives a euthanasia request, she must consider if she wants to fulfil that request. Research shows that physicians vary in their attitudes toward accepting or rejecting a euthanasia request [12]. These attitudes are partly dependent on the moral values of physicians. A moral value refers to what a physician deems important in performing the practice of medicine.

The aim of this study is to explore GPs’ experiences of euthanasia requests and the moral values that influence dealing with the request. The study is guided by the research question ‘Which moral values play a role for GPs when dealing with requests for euthanasia?’.

## Method section

### Design and recruitment

The study took a qualitative approach, consisting of semi-structured interviews with GPs involved in decision making regarding euthanasia.

GPs were recruited through purposive and snowball sampling. GPs were included if they had received euthanasia requests and followed the procedure as outlined in law. First, GPs were recruited by a physician, who had a role as an independent consultant (SCEN physician) in euthanasia request cases. Included GPs subsequently recommended others who fulfilled the inclusion criteria. None of the approached respondents refused to participate, nor were any interviews repeated. In this way, nine GPs were included in the study.

### Data collection

A topic guide was developed based on existing literature and the research question (see Box 1).

**Box 1:** Topic guide for GP interviews

**Topic guide**
Experiences with the last phase of life of patients
Experiences with euthanasia (including how many times and the reasons for the requests)
Dilemmas experienced when confronted with a euthanasia request
Actions taken after an approved euthanasia request
Communication with the patient’s family
Contacts and communication with other professionals during the euthanasia process

The interviews were carried out by a female PhD student (MvZ), trained in qualitative research interview techniques. The interviewer presented herself as having no strong moral views on the conduct of euthanasia, but wanted to explore the practice. The interviews were conducted in 2011 at the participant’s home or workplace, with nobody else present and lasted approximately one hour. Field notes were made directly after each interview and were used to inform the research team. The interviews were audio-recorded with the permission of the physicians and transcribed verbatim. Participants were informed in advance and at the start of the interview about the aims of the research and that their anonymity would be guaranteed.

### Analysis

Data analysis was carried out by two researchers (MvZ, FdB) following a three step inductive thematic approach [2]. First, the interview with the most detailed information was coded independently by the researchers, after which they compared their codes. A coding tree was drawn up with the codes on which they agreed. This coding tree was used to analyze the second interview, after which the same procedure of comparison of the codes took place and new codes were added. This process continued until the sixth interview. Field notes were referred to aid interpretation.

Second, codes were grouped into distinct themes reflecting perspectives of the participants on topics discussed during the interviews. The themes were discussed in group meetings (MvZ, FB, GW), which led to a selection of themes. Next, the quotations within the theme ‘physician values regarding end-of life’ were further analyzed by categorizing the quotes into subthemes in order to give more precisian and clarity to this theme [2, p. 63]. In the process of categorization, a list of twenty values related to end-of-life care was used for comparison. The list was based on two sources: literature on values in medical ethics and a value list of Movision [9]. It consisted of moral values such as autonomy, benevolence, empathy, gratefulness, hope, justice, quality of life, respectfulness, trustworthiness. Two researchers (MvZ, FdB) independently categorized each quote in relation to the selected values, after which they discussed the categorization of the quotes. The final categorization of values and related quotes was discussed in meetings among four researchers (MvZ, FdB, GW and NE), until consensus was reached. MvZ and FdB coded the data from the remaining three interviews according to the values table, and developed the final value themes collaboratively with GW and NE. No software program was used to analyze the data. For reporting, the Consolidated criteria for reporting qualitative research (COREQ) were followed [17].

## Results

Nine GPs were interviewed, males as well as females, with an age range from 35 to 65 years, some with a practice in a rural area and others in an urban area. Some GPs were religious; others described themselves as non-religious. All had performed euthanasia at least once, apart from one young male participant, who had been involved in a euthanasia trajectory in which the patient died naturally before euthanasia could be performed. The cases which the participants described were not disputed cases; they were not related to for instance dementia or psychiatric diseases. The moral values which participants mentioned have been grouped in four main categories: values related to: 1) the patient; 2) the family; 3) the physician; and 4) life and death.

1. Values concerning the patient

Participants mentioned the following values concerning the patient: autonomy, openness, authenticity; they also mentioned the importance of the relationship with the patient.

### Autonomy

Participants attached great importance to the patient taking control of the final phase of life, including the request for euthanasia.

“[t]his was a man who was led by his wife all his life and this was one of the few things in which he took action himself from the start. That was very important to him.” (Doctor 5, >50)

### Openness

The participants expected that patients should be open about their own wishes, open to other options, and open to understanding of what they ask from the physician.

“I can also sense why she makes that request, and I see that she knows what she is asking and what it means to the doctor”. (D8, >50)

### Authenticity

Participants stressed that the patient’s request should be authentic. This implies that it should express the patient’s experience of being unable to endure the situation.

“What makes that someone really cannot tolerate it […]; what makes it so bad for someone.” (D5)

Authenticity was also related to the patient’s ability to reach narrative closure.

“It is done yes well, and I am leaving now. And I will look down upon you from above, and I will give you my blessing.” (D2, >50, quoting the patient’s goodbye words to her family)

### The relationship with the patient

Participants stated that an established relationship with the patient is important in dealing with a euthanasia request.

“I didn’t treat her at all, I hardly ever saw her. And then suddenly, I got that request for euthanasia. […] That didn’t feel right for me at all.” (D7, <50)

Going through a process of granting a euthanasia request can also deepen the patient-physician relationship.

“A very intensive but also fulfilling process […] because you come into contact with patients much more closely and intensively than what you normally have with a back problem or a cold.” (D8)

2. Values concerning the family

Participants also emphasized the value of a good relationship with the family. This includes involving the family and caring for the family.

### Involving the family

Although the patient comes first, the family should also be involved in the decision-making process. If the family opposes, this requires attention and time.

“I cannot imagine performing euthanasia on a patient whose wife does not support it. I cannot imagine anything like that. Then I would think we’d just have to put it off for a few weeks and we’d have to talk about it again.” (D8).

### Caring for the family

Participants stressed that the family also needs attention and support after the death of the patient.

“With euthanasia, then everything is settled and done at some point, with all the papers well, ok then, then you sit down for a while [with the family] and ask: Can you talk about that period, what has it been like?” (D7)

3. Values concerning the physician

Participants described the following values related to themselves as physician: autonomy, responsibility, understanding, and relief of suffering.

### Autonomy

Participants stressed that it must be their own decision regarding whether or not to grant a request for euthanasia.

“Well, of course I have to feel for myself: this is a good decision. I can support this as a physician.” (D8)

Participants emphasized that the patient needs to convince them about the reason for the request, which can take time.

“You have to have a damned good reason for that if you want it [euthanasia].” (D2)

"I said: "Well, I am not ready yet.” And then we discussed it again, and again, and finally I thought, now I find it palpable. Then I can go along with it." (D7)

### Responsibility

Participants said that in deciding to go along with the request, they take responsibility for the process.

“These are decisions, a difficult point in which you have a unique influence in which every physician will be different.” (D4, >50)

Deciding to go along with a request for euthanasia is regarded as impactful.

“It is the ultimate thing you do in the life of another.” (D3, >50 )

### Understanding

An important value for participants is understanding of the patient, which can develop gradually.

“And as a physician you have to understand why someone wants it, otherwise you will do it against your feelings. If you think it is not right, then it can't be.” (D5)

“And at some point, I could get into it and I was completely on the same line with her, and I thought: it is probably the best for her to perform euthanasia. So, then I wanted to help her with it." (D6 <50)

### Relief of suffering

The decision to go along with a request for euthanasia is motivated by the value of relieving suffering.

“But at some point, you have nothing left to alleviate that suffering. Then it's a relief that that suffering can stop.” (D7).

“What's first is that you're going to prevent a lot of suffering. That is why I am doing it. And that that can only be done by letting someone die in the end. That is of course very extreme and very tough.” (D8)

Relief of suffering is not only related to helping the patient to die, but also entails making sure that the process of euthanasia is peaceful.

“Ensuring that things go as smoothly as possible. With as few complications as possible. As little trouble as possible.” (D6)

4. Values concerning life and death

Participants mention values related to life and death: life as an intrinsic good; and a peaceful death.

### Life as an intrinsic good

Euthanasia implies actively ending the life of a patient. That goes against the value of life as an intrinsic good.

“To respect life. Yes, not because of faith. I mean, we all have the urge to live, we all want to live and not to die." (D3)

Some participants described performing euthanasia as fearsome and exceptional.

“So, my first reaction is always a fright. Although it is as clear as what, I am shocked." (D3)

Regardless of the exceptional nature of the action, none of the participants expressed feelings of regret.

“But I am nevertheless happy to be able to do it. I am glad that I can do it. I never had the feeling afterwards: I shouldn't have done this.” (D8)

### A peaceful death

In the participants' decisions about euthanasia, their views on what constitutes a good death played a major role. For the participants, a good death means that the patient does not die in solitude but peacefully within the family circle, often at home.

“There you see a man and his daughters, who comfort their father with washcloths and caressing his head and reading a favorite poem. Then you almost get tears in your eyes. If you must die, then in those circumstances, in those warm conditions.” (D3)

“You try to prevent someone from ending up in hospital in their final phase of life. You don't want to die in the hospital, you want that at home.” (D7).

## Discussion

In dealing with a euthanasia request, the participants in this study were guided by values concerning 1) the patient; 2) the family; 3) the physician and 4) life and death.

It turned out that patient autonomy was a necessary condition for meeting the euthanasia request, but not a sufficient one. There also had to be a good relationship with the patient and the family. Also, values related to the role of the physician were mentioned. Essential for the participants was respect for the intrinsic value of life. Yet, participants did not regret having performed euthanasia afterwards, especially if it contributed to a peaceful death.

### Various values influence physician’s decision-making regarding a euthanasia request

The study shows that participants endorse various values in relation to receiving a euthanasia request. This may explain why physicians have different attitudes toward accepting or rejecting a euthanasia request [12]. Participants try to deal with and balance different values. For our participants, this does not result in a general attitude for or against performing euthanasia, but in a specific decision on an individual euthanasia request. The relief that the participants experienced after performing euthanasia was the result of weighing and deliberating different options.

Proponents of patient autonomy perceive euthanasia as a form of patient self-determination. [8]. According to Kennedy [7], since the 1960’s the moral value of respect for autonomy has become increasingly important in the Netherlands. For the participants, respecting patient’s autonomy is clearly important, but openness and authenticity of the patient, relationship with the patient and the family, values related to their professional role, and the intrinsic value of life and a peaceful death were also influential in dealing with euthanasia requests.

Respect for the patient's autonomy meant more for the participants than following patient’s wishes, as can be seen in the emphasis on openness and authenticity. The concept of autonomy involved is not self-determination, but relational autonomy [18], emphasizing that all those involved in a euthanasia request should participate in the decision-making process. For the participants, a good death means not dying in solitude and including the family to participate in the last period of the patient’s life, resulting in a peaceful death. This is in line with the findings of Ten Cate, who stresses the importance for physicians of “acceptance and resignation, being supported by loved ones, harmony, and being at home.” [16, p. 73]. If the family had difficulty in accepting the coming death of the patient, the participants tried to take time to get the family on board. Participants mentioned satisfaction when the family showed gratitude afterwards.

The interpretation of patient autonomy in terms of openness, authenticity and relationships is in line with the deliberative model of Emanuel and Emanuel [4]. They have distinguished four models of the physician–patient relationship: 1) the paternalistic, 2) the informative, 3) the interpretative and 4) the deliberative model. Their preference is for the latter model because the values of both the physician and the patient play an equally important role. The ideal is to decide together. Emanuel and Emanuel [4] argue that too much emphasis on the patient's role is undesirable, as is too much emphasis on the role of the physician. They indicate that the values of the physician are indispensable: "… physician values are relevant to patients and do inform their choice of a physician". Kouwenhoven et al. [8] and Van der Geest and Satalkar [5] have also questioned the dominant role of patient autonomy in the sense of self-determination in the Dutch debate on euthanasia, and have emphasized other concepts of autonomy such as autonomy as an ideal and as a social skill without losing one’s own control.

Another pivotal value is physician autonomy. This study shows that physicians should have room to make their own decisions, which can take time. Also, responsibility, understanding and relief of suffering are important values. These values are related to ethics of care [18].

The participants were aware that euthanasia entails killing a human being, which made them reluctant to simply go along with the patient's request. To better understand this outcome of the study, it can be helpful to refer to Dworkin’s analysis of the value ‘sanctity of life’ [3, p. 68–101]. According to Dworkin, human life is subjectively, instrumentally and intrinsically valuable. "Something is intrinsic valuable if its value is independent of what people happen to enjoy or want or need or what is good for them." (p. 71) Ten Have et al. (1998) explain that this specifically relates to human life. Dworkin describes that the value that life has for the person himself, is the subjective value. "So if we say that life has lost its value to someone [… …], we are treating that life in a subjective way". (p. 73) The instrumental value means that life is a mean of achieving other goals such as autonomy. "After all, it wouldn't be the life that really matters, but the goals we hope to achieve." (Ten Have et al. 1998, p. 63) A patient may believe that continuing life is not worthwhile anymore because one suffers too much, experiences hopelessness and feels not being able to contribute to others. Subjectively and instrumentally, the patient may think it is not useful to extend life, which leads to the request of euthanasia. Yet, the intrinsic value of life is something different. Participants were able to empathize with the patient's wishes, but could not simply grant the request for euthanasia, because they regard it as a violation of the intrinsic value of life. This does not mean that euthanasia cannot be performed. Other values, specifically ending suffering and fostering a good death, can override the moral duty to preserve life.

The main contribution of this study is the attention for the moral values of physicians in case of a euthanasia request. This study adds a new perspective to the Dutch public debate on euthanasia by showing the moral values that physicians take into account and weigh when considering a request for euthanasia.

### Limitations of the research

This study is based on nine interviews with physicians who were selected because they had dealt with a euthanasia request in their medical practice. This is a small number, which makes it difficult to transfer the results to Dutch medical practices and their dealing with euthanasia requests. On the other hand, the interview data of this study have been carefully analyzed and moral values are difficult to quantify. Studying a limited number of cases can stimulate others to add other perspectives to it in larger studies. Also, the results correspond to those of other studies, which were not used to guide the analysis. Although the data were collected several years ago, we do not expect major changes, as the focus is on fundamental values, which do not change overnight.

### Recommendations for practice

The results indicate that euthanasia needs careful preparation – as expressed in Dutch law – and next to it – on the level of practice – a deliberative model of the physician–patient relationship which involves respect for the different perspectives and careful communication with different persons involved. Although, the regulations of permitting euthanasia in the Netherlands are carefully worked out and monitored, tensions between values in medical practice require further attention. Therefore, we recommend more research on moral dilemmas that physicians face when being confronted with a euthanasia request and their needs in dealing with these dilemmas.

## Conclusion

The study shows that the participants consider autonomy to be an important value, but that a patient’s request in itself is not sufficient for deciding to perform euthanasia. Participants expressed that it is necessary to be connected to the patient and the family, to act in accordance with values related to their professional role and to take into account values related to life and death. The awareness of going against the intrinsic value of life remains important, even when euthanasia may result in a feeling of relief or gratitude afterwards.

## Data Availability

The datasets used and/or analyzed are available from the corresponding author on reasonable request.

## Abbreviations

GP: General Practitioner
SCEN: Support and Consultation with Euthanasia in the Netherlands
COREQ: Consolidated Criteria for Reporting Qualitative Research
